# CT Angiography in Patients Referred for Invasive Coronary Angiography: A Single Large-Volume Tertiary Center Experience

**DOI:** 10.3390/jcm14176211

**Published:** 2025-09-03

**Authors:** Migena Disha, Legate Philip, Daniel Dumitrescu, Volker Rudolph, Regine Brinkmann, Mohamed Ayoub

**Affiliations:** 1 Department of General and Interventional Cardiology/Angiology, Heart and Diabetes Center NRW, Ruhr University Bochum, 32545 Bad Oeynhausen, Germany; ddumitrescu@hdz-nrw.de (D.D.); vrudolph@hdz-nrw.de (V.R.); rbrinkmann@hdz-nrw.de (R.B.); mayoub@hdz-nrw.de (M.A.); 2 Porthsmouth Hsopitals University NHS Trust-Portsmouth, Portsmouth PO6 3LY, UK; legatephilip@doctors.org.uk

**Keywords:** computed tomography, cardiovascular disease, cardiac catheter, cardiovascular risk factors, non-invasive

## Abstract

**Highlights:**

Coronary CT angiography (CTCA) is a safe and effective non-invasive diagnostic tool for evaluating coronary artery disease (CAD) in patients with low to intermediate pre-test probability (PTP), consistent with current European Society of Cardiology (ESC) guidelines.

**What are the main findings?**

- In a real-world, single-center study, CTCA demonstrated similar long-term clinical outcomes compared to invasive coronary angiography (ICA), with no significant differences in adverse events or functional outcomes assessed by stress echocardiography.
- CTCA demonstrated a high negative predictive value (89%), confirming its reliability in safely ruling out CAD and potentially avoiding unnecessary invasive procedures.
- CTCA significantly reduced diagnostic time and hospital stay compared to ICA (4.7 vs. 20.2 h, *p* < 0.0001), highlighting its potential for efficient outpatient workflows.
- Despite a higher mean radiation dose in the CTCA group (2.3 mSv vs. 1.5 mSv, *p* = 0.03), no procedure-related complications occurred in this group, unlike the ICA arm.
- Performance metrics of CTCA (sensitivity 75%, specificity 77%) were slightly lower than those reported in multicenter trials, possibly due to the small sample size and single-center design.

**What are the implications to the main findigs?**

- Findings support broader implementation of CTCA as a first-line diagnostic tool in stable CAD, especially following its recent reimbursement approval in Germany (2024).

**Abstract:**

**Background/Objectives:** Coronary artery disease (CAD) is a major cause of mortality worldwide, accounting for 7.3% of all deaths in Germany. Invasive coronary angiography (ICA) remains the gold standard for diagnosing CAD, yet coronary computed tomography angiography (CTCA) is gaining recognition as a non-invasive alternative. Recent clinical trials have confirmed CTCA’s diagnostic accuracy, leading to its inclusion in the 2019 European Society of Cardiology (ESC) guidelines. Despite this, its adoption in Germany has been slow. **Methods:** This single-center, non-randomized study at the Heart and Diabetes Center North Rhine-Westphalia (HDZ NRW) evaluated CTCA’s safety and diagnostic performance. We included patients with low to intermediate pre-test probability (PTP) referred for cardiac catheterization between 2019 and 2022. The primary outcome was the change in the Wall Motion Score Index (ΔWMSI), with a threshold of 0.37 indicating significant mortality risk. Secondary outcomes included cardiovascular mortality, myocardial infarction, angina at follow-up, and myocardial revascularization procedures. **Results:** A total of 100 patients were enrolled; 30 underwent CTCA, and 70 had ICA. The mean patient age was 63 years, with 33% female. Of the 63 patients who completed follow-up (41 ICA, 22 CTCA), no significant differences in cardiovascular outcomes or mortality were observed. CTCA effectively ruled out CAD in low-risk patients, with a sensitivity of 75% and specificity of 77%. CTCA was faster (4.7 vs. 20.2 h) but had a higher radiation dose (2.3 vs. 1.5 mSv). **Conclusions:** CTCA is a viable, non-invasive alternative for diagnosing low- to intermediate-risk CAD patients. Further studies are needed to confirm its clinical benefits.

## 1. Introduction

Cardiovascular diseases (CVD), particularly coronary artery disease (CAD), are a leading cause of morbidity and mortality worldwide. In Germany, CVD accounts for 40% of all deaths [1], with approximately 7.3% attributable to CAD [1]. Given these statistics, the improvement of coronary diagnostic modalities has been a continuous focus. While invasive coronary angiography (ICA) has traditionally served as the reference standard for diagnosing CAD, advancements in imaging techniques, particularly coronary computed tomography angiography (CTCA), have demonstrated promising results in terms of safety and cost-effectiveness.

Clinical trials such as PROMISE [2], Scot-Heart [3], and DISCHARGE [4] have validated the efficacy of CTCA, establishing it as a reliable non-invasive imaging modality for patients with a low to intermediate pre-test probability of CAD. In 2019, the European Society of Cardiology (ESC) updated its guidelines [5] for stable CAD, recommending CTCA as the preferred imaging tool for patients with chronic CAD.

Despite this, CTCA has not yet been routinely adopted as the primary non-invasive diagnostic modality for patients with stable angina in Germany. Since January 2024, public health insurance companies in Germany have allowed CTCA as a routine diagnostic modality for patients pre-diagnosed with CAD who have intermediate Pre-test Probability (PTP) [6]. However, referring cardiologists still usually recommend an ICA to these patients.

Our study aims to evaluate the safety and reliability of coronary CT angiography as a routine diagnostic modality for patients with low or intermediate risk of coronary artery disease, comparing it to the reference method, invasive coronary angiography (ICA), in a real-life setting.

## 2. Methods

### 2.1. Patient Recruitment

**Figure 1:**
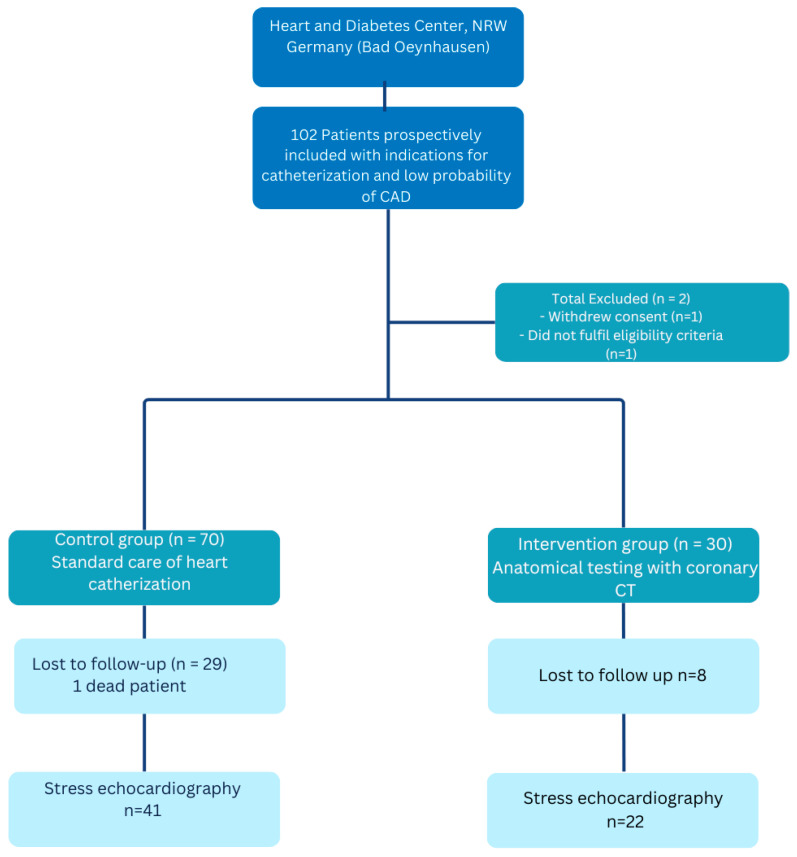
Methods flow chart comparing CTCA to ICA. Flow diagram of patient inclusion, group allocation, and follow-up in a prospective comparison of coronary computed tomography angiography (CTCA) versus standard invasive coronary angiography (ICA). A total of 102 patients with clinical indications for coronary catheterization and low to intermediate pre-test probability of coronary artery disease were prospectively enrolled at the Heart and Diabetes Center, NRW, Germany. Two patients were excluded (one withdrew consent, one did not meet eligibility criteria). The remaining 100 patients were allocated to either the control group receiving standard ICA (n = 70) or the intervention group undergoing CTCA-based anatomical testing (n = 30). Follow-up stress echocardiography was completed by 41 patients in the control (ICA) group (29 lost to follow-up, including one death) and 22 patients in the intervention (CTCA) group (8 lost to follow-up).

Figure 1 shows the flow chart of patient selection into the study. All consecutive patients referred for cardiac catheterization between November 2019 and April 2022 were considered for enrolment in the study. This was a single-center, non-randomized trial conducted at the cardiology department of the Heart and Diabetes Center North Rhine-Westphalia (HDZ NRW) in Bad Oeynhausen.

Patients were deemed eligible for inclusion if they had a low to intermediate pre-test probability for coronary artery disease (CAD), categorized as 15–50% according to the 2019 European Society of Cardiology (ESC) guidelines for stable CAD [5]. We excluded individuals with a prior diagnosis of CAD, those aged over 75 years presenting with typical angina pectoris, and patients admitted primarily for evaluation of valve disease.

The study was approved on 4 October 2019 by the ethics committee of the medicine faculty, Ruhr University Bochum (RUB), in Germany.

### 2.2. Study Design

We intended to assign patients in a 1:2 ratio to either the CT coronary angiography (CTCA) group or the control group, which underwent invasive coronary angiography. All patients participating in both groups were offered a follow-up procedure of stress echocardiography after a minimum interval of six months as a surrogate parameter for long-term outcome regarding myocardial function. This was based on the study findings of Sicari et al. [7], showing favorable patient outcomes with normal echocardiographic stress tests.

CT arm: All diagnostics conducted within the CT arm of the study were carried out by the radiology team at HDZ NRW. The team utilized a 320-slice Aquilion One Genesis (CanonMedical Systems, Otawara, Japan) for the CT scans. After consultations with radiologists, the severity of coronary artery stenoses and calcifications was classified according to the Coronary Artery Disease Reporting and Data System (CAD-RADS) score [8]. Patients presenting an Agatston score of 0, indicating the absence of calcifications, or those without stenoses, were discharged without any further recommendations. In instances characterized by significant stenoses, a heart team consisting of the radiologists and experienced cardiologists decided in cases of elevated CAD-RADS scores or ambiguous findings. We performed either another non-invasive method, like stress myocardial Scintigraphy or the standardized invasive catheter angiography.

ICA arm: Coronary interventionalists with extensive experience at our center conducted and evaluated invasive coronary angiograms. When a lesion was detected, the decision to either treat it or assess its hemodynamic implications was left to the discretion of the operators. In general, mild lesions were managed conservatively, while moderate lesions underwent further evaluation through physiological assessment or intracoronary imaging. Significant lesions were addressed either by percutaneous intervention or by referring the patient for coronary artery bypass grafting (CABG).

All patients in both groups were offered a follow-up stress echocardiography procedure after a minimum interval of six months. During follow-up, participants underwent brief interviews to discuss their symptoms prior to stress echocardiography. The assessment of wall motion in the left ventricle was conducted at rest, during stress, and at the conclusion of the test. The Wall Motion Score Index (WMSI) was computed using the GE EchoPac PC v204 Software only (United States of America), based on a 16-segment left ventricular map. Each segment was evaluated according to the following criteria: normokinesia (1 point), hypokinesia (2 points), akinesia (3 points), and dyskinesia (4 points) [9]. The WMSI was derived by dividing the total of the segmental scores by the overall number of myocardial segments (16). A WMSI of 1 (16/16) signifies normal ventricular function. Patients exhibiting symptoms indicative of typical angina during the stress test, or demonstrating positive findings following stress echocardiography, were referred for consultation with invasive cardiologists. This referral applied particularly to cases involving left ventricular systolic dysfunction, as evidenced by a Wall Motion Score Index (ΔWMSI) difference exceeding 0.37, or showing ischemic changes on the electrocardiogram (ECG). Subsequently, these patients underwent additional diagnostic interventions, including invasive coronary angiography (ICA).

### 2.3. Endpoints

The primary outcome of this study was the change in the Wall Motion Score Index (ΔWSMI), which was determined by calculating the difference in the WMSI from stress testing to resting conditions. A ΔWSMI score exceeding 0.37 was considered significant in predicting cardiac mortality during long-term follow-up, as detailed in the study by Sicari et al. [7].

Secondary outcomes included mortality, myocardial infarction, hospital admissions due to unstable angina or angina at follow-up, and myocardial revascularization procedures.

## 3. Statistical Analyses

As presented in Table 1, a balance test was conducted to confirm that both groups were comparable concerning baseline characteristics, cardiac risk factors, symptoms, and pre-diagnostic modalities. Continuous variables were analyzed utilizing *t*-tests, while categorical variables were evaluated using chi-square tests. The statistical analyses were carried out using IBM SPSS Statistics version 29.0 (Chicago, IL, USA) and Excel version 16.87, Microsoft Office 2024.

## 4. Results

### 4.1. Study Population

Between November 2019 and April 2022, a total of 102 consecutive patients were enrolled at our center. Of these, two patients withdrew from the study (Figure 1). Among the remaining 100 patients, 30 received a diagnosis through computed tomography (CT), while 70 underwent conventional treatment involving invasive coronary angiography (ICA). A total of 63 patients completed the follow-up, which had a median duration of 10 months.

### 4.2. Baseline Characteristics

Table 1 provides an overview of the baseline characteristics and results of the balance tests for the coronary CT and ICA groups. The mean age of patients in the coronary CT group was 63 ± 11.6 years, compared to 65 ± 9.4 years in the ICA group, indicating no statistically significant difference (*p* = 0.26). The distribution of sex was comparable across both groups, with 67% of the participants being male.

The principal cardiac risk factors, including hypertension, diabetes, and smoking, were similar between the two groups, with hypertension being the most prevalent risk factor noted.

The symptom most frequently reported was angina pectoris, which was observed in 60% of patients in the coronary CT group and in 54.3% of those in the ICA group, again showing no significant difference (*p* = 0.59). In summary, there were no statistically significant differences identified between the two groups for any of the assessed variables.

### 4.3. Diagnostic Results

#### 4.3.1. CTCA Arm

**Figure 2:**
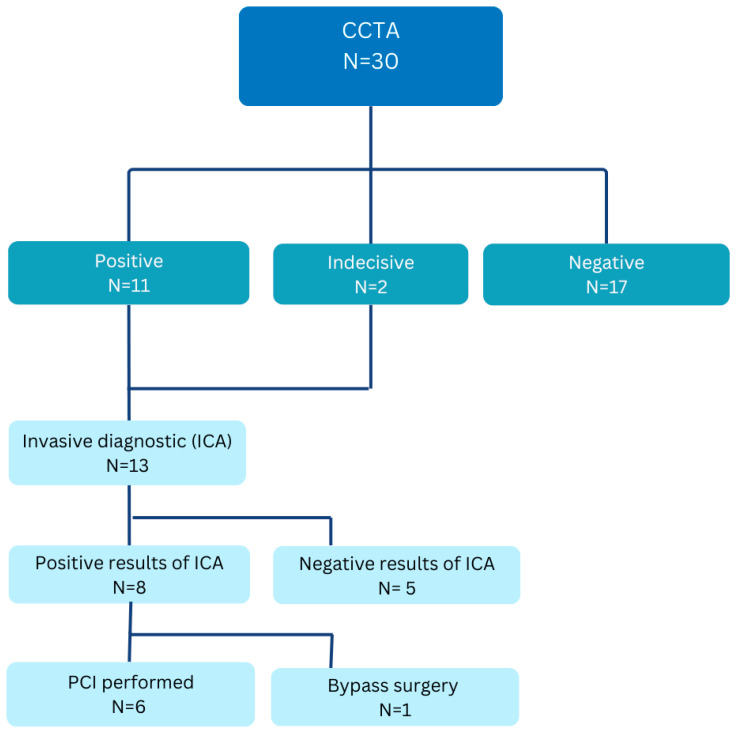
Diagnostic flow chart from the CTCA group. Diagnostic pathway of patients in the CTCA group. Following coronary computed tomography angiography (CTCA), 11 patients had positive findings, 2 had inconclusive results, and 17 had negative findings. Patients with positive or inconclusive results proceeded to invasive coronary angiography (ICA). Among those, 8 had positive findings on ICA, leading to percutaneous coronary intervention (PCI) in 7 patients and coronary artery bypass grafting (CABG) in 1 patient. Patients with negative CTCA results were managed non-invasively.

Figure 2 shows the pathological CTCA results and the following diagnostic procedures or treatment.

In our cohort, all patients exhibiting a non-zero CalcScore or stenoses classified by the CADRadsScore underwent further evaluation. The CalcScores within this cohort exhibited considerable variability, ranging from a minimum of 0 to a maximum of 1119. The positive CalcScore results were referred for invasive coronary angiography (ICA). Conversely, patients presenting a CalcScore of 0 or lacking stenotic lesions were classified as non-pathological and subsequently discharged without further recommendations. Notably, one patient presented with a low CADRadsScore of 32 and no evidence of stenoses. This patient underwent myocardial scintigraphy as a functional assessment, which indicated no signs of ischemia.

A total of 11 out of 30 patients (37%) exhibited pathological results on their CT scans in the CTCA arm. Furthermore, two patients (6%) presented with image artifacts, resulting in inconclusive findings. Given their typical symptoms indicative of coronary artery disease, we proceeded with invasive coronary angiography (ICA) for both individuals. An invasive coronary angiography (ICA) was conducted for all 13 patients. Among these, eight patients (27%) were later confirmed to have pathological coronary disease through ICA, as illustrated in Figure 2. Of the eight patients identified with coronary disease, six underwent interventional percutaneous coronary intervention (PCI), one received coronary artery bypass graft surgery (CABG), and one patient was diagnosed with moderate coronary artery stenosis but did not require any intervention. The remaining five patients did not exhibit any coronary pathologies and, consequently, did not necessitate further procedures. Additionally, 17 out of 30 patients (57%) in the CTCA arm either had a coronary calcium score of 0 or did not present with any stenoses as diagnosed by the CTCA. Consequently, these patients were discharged without the need for further procedures or recommendations.

**Figure 3:**
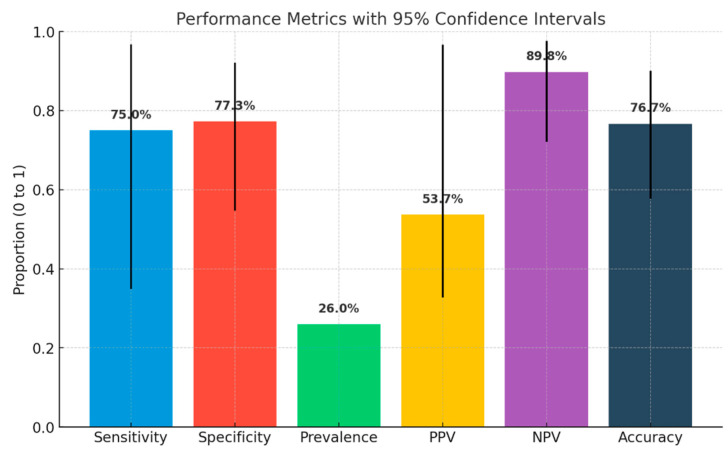
Sensitivity, specificity, positive predictive value, and negative predictive value of CTCA. Sensitivity, specificity, positive predictive value (PPV), and negative predictive value (NPV) of coronary computed tomography angiography (CTCA). This figure summarizes the diagnostic performance of CTCA for detecting significant coronary artery disease, using invasive coronary angiography (ICA) as the reference standard.

To evaluate the reliability of the CTCA as a non-invasive method, we calculated, as presented in Figure 3 the results for sensitivity, specificity, positive predictive value (PPV), and negative predictive value (NPV). A diagnostic test evaluation was conducted on a subgroup of patients from the CTCA cohort who demonstrated positive results for CAD, using ICA as the comparative reference standard. According to the data presented in Figure 3, the sensitivity of computed tomography coronary angiography (CTCA) was recorded at 75%, while the specificity was established at 77.27%. The PPV for CTCA was determined to be 53%.

#### 4.3.2. ICA Arm

**Figure 4:**
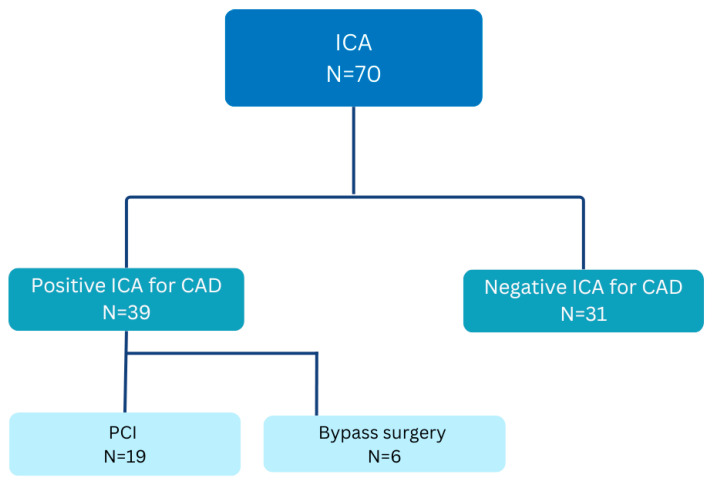
Diagnostic flow chart from the ICA group. Diagnostic pathway of patients in the invasive coronary angiography (ICA) group. All 70 patients underwent ICA as the initial diagnostic approach. Coronary artery disease (CAD) was confirmed in 39 patients, leading to percutaneous coronary intervention (PCI) in 19 patients and coronary artery bypass grafting (CABG) in 6 patients. No significant CAD was found in 31 patients.

In the control group undergoing invasive coronary angiography (ICA), 39 out of 70 patients (55%) received a diagnosis of coronary artery disease. Among these patients, 19 (27%) proceeded to undergo percutaneous coronary intervention (PCI), while 6 (8%) required cardiac bypass surgery, as illustrated in Figure 4. The remaining 31 patients exhibited no evidence of coronary pathology and were subsequently discharged without further recommendations.

### 4.4. Follow-Up and Endpoint Measures

Table 2 provides an overview of the coronary findings and procedural outcomes. No significant differences were analyzed between two groups.

Table 3 presents the outcomes observed at the follow-up, which had a median duration of 10 months. There were no significant differences in the outcomes between the two groups.

**Figure 5:**
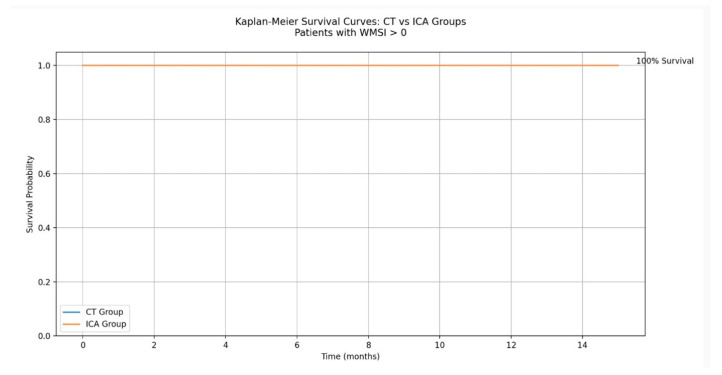
Kaplan–Meier survival analysis of the Wall Motion Score Index (WMSI). Kaplan–Meier survival analysis based on the Wall Motion Score Index (WMSI). Patients were stratified according to their ΔWMSI (change in WMSI), with a threshold of 0.37 identified as both statistically and clinically relevant. No significant difference in survival was observed between the groups above and below this cutoff. Both groups demonstrated a survival rate of 1.0 during the follow-up period, indicating no prognostic discrimination by ΔWMSI in this cohort.

The primary outcome assessed was the difference in the Wall Motion Score Index (∆WMSI), from stress to rest, comparing results at follow-up with those at admission. Among the 63 patients available for follow-up, none demonstrated a ∆WMSI exceeding 0.37 or exhibited signs of ischemia based on the findings from the stress echocardiography. The survival analysis of the WMSI is depicted in Figure 5, indicating a survival rate of 1, which signifies that no changes in WMSI were detected in either group at the time of follow-up.

In relation to secondary outcomes, one patient in the ICA group died. This particular patient had a history of kidney transplantation several years prior, and no conclusive cause of death was determined. Moreover, only one patient in the CT group reported experiencing angina during the follow-up assessment. There were no recorded instances of myocardial infarction or revascularization at the follow-up evaluation.

We conducted a *t*-test analysis to evaluate further possible differences, based on several parameters like effective radiation dose, contrast agent quantity, overall diagnostic time, admission time, and either inpatient or ambulant care, between the two groups.

The diagnostic time was assessed based on the documented records of the respective teams. For the CTCA group, the overall radiation exposure duration was considered as the diagnostic time. Conversely, for the ICA group, the diagnostic time was defined as the interval from puncture to sheath removal. Additionally, the total admission time for each patient was calculated from admission to discharge, as documented in the medical records. Our findings indicated no significant differences between the groups concerning the quantity of contrast dye administered or the overall diagnostic time (Table 4).

Significant differences were observed in the effective radiation dose, with a mean dose of 2.3 mSv for the CTCA group compared to 1.5 mSv for the ICA group (*p* = 0.03). Furthermore, the mean overall diagnostic time was 4.7 h for the CTCA group, in contrast to 20.2 h for the ICA group (*p* < 0.0001).

## 5. Discussion and Study Limitations

In a real-life setting conducted at an experienced tertiary care center, we evaluated the diagnostic efficacy of less invasive computed tomography coronary angiography (CTCA) in comparison to invasive coronary angiography (ICA). The results of our study indicate no significant differences in adverse cardiovascular events, mortality rates, or clinical manifestations, including the Wall Motion Score Index and reported episodes of angina pectoris, between the two diagnostic modalities.

We performed a survival analysis using the Kaplan–Meier curve comparing the two methods based on the delta WMSI.

We presented this analysis in Figure 4, indicating no significant (HR 1, CI 95%) differences between the two groups at follow-up, suggesting that routine application of CTCA offers an effective alternative for diagnosing CAD in patients with low to intermediate pre-test probability (PTP). Our findings align with the results from trials such as DISCHARGE [4] and Scot-Heart [3], which highlight the non-inferiority of CTCA as a diagnostic tool compared to other non-invasive functional methods and invasive coronary angiography (ICA). Figure 5 indicates that CTCA possesses a strong negative predictive value of 89%, consistent with findings from the PROMISE [2] trial. This supports the utilization of CTCA as a reliable method for ruling out CAD, thereby meeting the expectations of both healthcare professionals and patients. The subgroup analysis in our study indicated that performance metrics were lower than anticipated, with a sensitivity of 75% and a specificity of 77%. Furthermore, the positive predictive value (PPV) in our study was measured at 53%, which is markedly below the general range of 64–92% documented in the existing literature [10]. These variations may be attributed to the relatively small cohort size within the CTCA group.

The average admission time for CTCA was recorded at 4.7 h, significantly shorter than the 20.2 h required for Invasive Catheterization Angiography (ICA). Furthermore, CTCA does not necessitate intensive patient monitoring following the procedure, in contrast to ICA. The CTCA group reported no complications, whereas the ICA group experienced a single incident of hematoma at the puncture site, with no subsequent deterioration in the patient’s condition. Additionally, randomized trials, such as DISCHARGE, have revealed that complication rates associated with Invasive Catheterization Angiography are significantly higher when compared to those associated with CTCA. The effective radiation dose was significantly higher calculated at the CTCA group compared to the ICA group. We did not find significant differences between the two groups regarding the amount of contrast agent used or the overall diagnostic time.

All participants in our study had no prior history of coronary artery disease. The most common presenting complaint was typical angina, reported by 60%, while approximately 50% cited dyspnoea as their primary reason for referral. Additionally, 21% were asymptomatic yet demonstrated abnormal electrocardiograms or stress test results, leading to referrals for further evaluation to exclude significant coronary artery disease.

The diagnosis and management of coronary artery disease are crucial topics in cardiology, as they significantly impact prognosis and morbidity. In cases of stable coronary artery disease (CAD), accurate diagnosis relies on patient-reported symptoms. However, variability in symptom reporting presents a challenge, as the absence or presence of atypical symptoms does not exclude significant coronary disease. Studies investigating the screening of asymptomatic patients for CAD have not shown clear advantages.

In light of these uncertainties, non-invasive tests play a vital role, especially for patients with low to intermediate pre-test probabilities. Stress tests, such as myocardial perfusion imaging (MPS) and stress echocardiography, are valuable for identifying prognostic indicators, although they have limitations, including the potential for false negative results in balanced ischemia and oversights in left main coronary disease, valvular heart disease, or reduced left ventricular function.

The European Society of Cardiology (ESC) has offered recommendations for coronary artery disease (CAD) and CT coronary angiography (CTCA) since 2019. Notably, starting in January 2024, patients in Germany with cardiological indications and public health insurance will be able to undergo CTCA at accredited centers. This change is expected to increase both the number of procedures performed and the certified centers available.

Moreover, it highlights the need for future multicenter trials to evaluate the cost-effectiveness of CTCA compared to invasive coronary angiography (ICA) in Germany. Currently, only 30 cardiac CT centers in Germany are recognized by the German Cardiac Society to train and certify cardiologists and radiologists in cardiac CT.

Our study has several important limitations. First, the small sample size may be underpowered to identify significant differences between groups. Additionally, 37 patients were lost to follow-up, primarily due to the impact of the COVID-19 pandemic. However, all patients were contacted via phone, and their symptoms were evaluated. Moreover, since our study was not randomized, we cannot exclude potential selection bias. The choice of some patients to undergo CT coronary angiography (CTCA) instead of invasive coronary angiography (ICA) may also have introduced a self-selection bias.

### Implications for Practice

Our study supports the assertion that CT coronary angiography (CTCA) is a safe and reliable non-invasive modality for the diagnosis or exclusion of coronary artery disease (CAD) when appropriate clinical indications are met. The future of non-invasive diagnostics appears promising for CT coronary angiography (CTCA), especially with the emergence of photon-counting CTCA technology, enabling a substantially higher image resolution. To further validate its effectiveness and its role in detecting atherosclerosis in patients with angina, there is a need for large-scale trials.

## Figures and Tables

**Table 1:** Baseline characteristics of patients. Baseline characteristics of the study population. This table presents socio-demographic data, cardiovascular risk factors, reported symptoms, and pre-diagnostic test findings of patients assigned to either the CTCA (computed tomography coronary angiography) group or the ICA (invasive coronary angiography) group. Continuous variables are reported as mean and standard deviation (SD), while categorical variables are expressed as numbers and percentages. Statistical comparisons between groups were performed using Student’s *t*-test or Chi-square test, as appropriate. No statistically significant differences were observed between the groups across measured parameters, indicating comparable baseline profiles.

**Socio-Demographic Variable**	**Description**	**CT (n = 30)** **Mean ± SD or n (%)**	**ICA (n = 70)** **Mean ± SD or n (%)**	***p*-Value**
Age (years)	Self-reported	63 ± 11.6	65 ± 9.4	0.26
Male sex		20 (66.7%)	47 (67.1%)	0.96
Height (cm)	Self-reported	174 ± 6.0	174 ± 9.4	0.98
Weight (kg)	Self-reported	87 ± 18.4	88.3 ± 18.6	0.43
Body mass index (kg/m^2^)	Calculated	28.8 ± 5.7	29.1 ± 5.8	0.83
Mean blood pressure (mmHg)	Ambulatory care	98.8 ± 12.1	103 ± 13.4	0.14
Heart rate (bpm)	Ambulatory care	68.3 ± 10.8	68.8 ± 11.8	0.84
Cardiac risk factors				
**Risk Factor**	**Description**	**CT (n = 30)**	**ICA (n = 70)**	***p*-Value**
Hypertension	Pre-diagnosed	23 (76.7%)	59 (84.3%)	0.36
Diabetes mellitus	Pre-diagnosed	8 (26.7%)	22 (31.4%)	0.63
Hypercholesterolemia	Pre-diagnosed	17 (56.7%)	42 (60.0%)	0.75
Current smoker	Self-reported	7 (23.3%)	23 (32.9%)	0.34
Ex-smoker	Self-reported	5 (16.7%)	16 (22.9%)	0.48
Adiposity	BMI-based	15 (50.0%)	30 (42.9%)	0.51
Family history of CAD	Self-reported	8 (26.7%)	24 (34.3%)	0.40
**Symptoms**				
**Symptom**	**Description**	**CT (n = 30)**	**ICA (n = 70)**	***p*-Value**
Angina	CCS 0–IV, self-reported	18 (60.0%)	38 (54.3%)	0.90
Dyspnea	Self-reported, primary/only symptom	4 (13.3%)	19 (27.1%)	0.13
**Pre-diagnostic tests**				
**Test**	**Description**	**CT (n = 30)**	**ICA (n = 70)**	***p*-Value**
Pathological ECG, extremity leads	Ambulatory care	4 (13.3%)	11 (15.7%)	0.76
Pathological ECG, chest leads	Ambulatory care	3 (10.0%)	5 (7.1%)	0.62
Pathological echocardiography (wall motion)	Referring cardiologist	2 (6.7%)	9 (12.9%)	0.36
Stress ECG performed	Referring cardiologist	9 (30.0%)	32 (45.7%)	0.14
Pathological stress ECG, extremity leads	Subgroup (performed only)	7 (77.8%)	18 (56.3%)	0.80
Pathological stress ECG, chest leads	Subgroup (performed only)	4 (44.4%)	13 (40.6%)	0.52
Stress echocardiography performed	Referring cardiologist	3 (10.0%)	8 (11.4%)	0.83
Pathological stress echocardiography	Subgroup (performed only)	3 (100.0%)	6 (75.0%)	0.81
Stress myocardial scintigraphy performed	Referring cardiologist	11 (36.7%)	31 (44.3%)	0.47
Pathological stress myocardial scintigraphy	Subgroup (performed only)	8 (72.7%)	30 (96.8%)	0.12

**Table 2:** Coronary artery diagnostic results and outcomes. Coronary artery diagnostic findings and procedural outcomes. This table summarizes the diagnostic and interventional results obtained during initial hospital admission for patients undergoing either computed tomography coronary angiography (CTCA) or invasive coronary angiography (ICA). Data include the prevalence and severity of coronary artery stenosis, adjunctive diagnostic modalities (IVUS, IFR/FFR), anatomical distribution of disease, and therapeutic interventions such as percutaneous coronary intervention (PCI) and coronary artery bypass grafting (CABG). Radiation exposure and contrast media volume are also reported. Continuous variables are presented as mean ± standard deviation, and categorical variables as number and percentage. Group comparisons were performed using *t*-tests or Chi-square tests, with corresponding *p*-values shown.

Variable	Description	CT (n = 30)	ICA (n = 70)	*p*-Value
Coronary stenoses (all)	Any degree	13 (43.3%)	41 (58.6%)	0.16
Moderate stenosis	75–90%	3 (10.0%)	16 (22.9%)	0.13
Severe stenosis	>90%	3 (10.0%)	18 (25.7%)	0.07
Chronic total occlusion (CTO)		2 (6.7%)	7 (10.0%)	0.59
RCA stenosis	ICA diagnosed	6 (46.2%)	22 (52.9%)	0.24
RCX stenosis	ICA diagnosed	5 (38.5%)	24 (57.8%)	0.07
LAD stenosis	ICA diagnosed	11 (84.6%)	29 (69.9%)	0.65
Left main stenosis	ICA diagnosed	1 (3.3%)	1 (1.4%)	0.53
Hemodynamically significant stenosis	ICA diagnosed	8 (26.7%)	28 (40.0%)	0.20
PCI performed	Intervention	5 (16.7%)	18 (25.7%)	0.32
CABG performed	Surgery	1 (3.3%)	7 (10.0%)	0.43
RCA PCI	Intervention	3 (10.0%)	9 (12.9%)	0.68
RCX PCI	Intervention	0	9 (12.9%)	0.04
LAD PCI	Intervention	4 (13.3%)	11 (15.7%)	0.76
Left main PCI	Intervention	1 (3.3%)	1 (1.4%)	0.53
Effective radiation dose (mSv)	Mean ± SD	2.33 ± 1.26	1.70 ± 1.68	0.04
Contrast volume (ml)	Mean ± SD (range)	55.5 ± 6.1 (50–70)	76.7 ± 35.4 (29–164)	<0.01

**Table 3:** Follow-up outcomes. Clinical outcomes during follow-up. This table presents follow-up outcomes (range: 6–31 months) for patients initially assessed via computed tomography coronary angiography (CTCA) or invasive coronary angiography (ICA). Outcomes include cardiovascular mortality, rehospitalization due to angina, stress echocardiography results, symptoms under stress (angina and dyspnea), and changes in wall motion score index (ΔWMSI). Continuous variables are expressed as mean ± standard deviation, and categorical outcomes are presented as numbers and percentages. Group comparisons were performed using *t*-tests or Chi-square tests as appropriate, with *p*-values reported.

Outcome	Description	CT (n = 30)	ICA (n = 70)	*p*-Value
Follow-up duration (months)	Mean ± SD	8.6 ± 2.7	12.2 ± 4.8	0.002
Cardiovascular deaths	Clinical	0	1 (non-angina)	–
Rehospitalization for angina	Clinical	1 (3.3%)	1 (1.4%)	0.90
Stress echocardiography follow-up	Clinical	22 (73.3%)	41 (58.6%)	0.16
Angina under stress	Clinical	1 (4.0%)	0	0.19
Dyspnea under stress	NYHA classification	14 (60.0%)	21 (51.2%)	0.72
ΔWMSI = 0	Calculated	22 (73.3%)	41 (58.6%)	0.27

**Table 4:** Subgroup analysis. Subgroup analysis comparing contrast dye volume, radiation exposure, and procedural time between CTCA and ICA. This table presents a comparative analysis between patients undergoing computed tomography coronary angiography (CTCA) and those receiving exclusive invasive coronary angiography (ICA). Variables assessed include contrast agent volume (ml), effective radiation dose (mSv), total admission time (hours), and diagnostic procedure time (minutes). Mean values are reported with standard deviations where applicable. Statistical comparisons were made using two-sided *t*-tests.

Variable	CT (Mean)	ICA (Mean)	*p*-Value
Contrast dye (mL)	55	66	0.09
Effective radiation dose (mSv)	2.3	1.5	0.03
Admission time (hours)	4.8 ± 0.2	20.2 ± 11.1	<0.0001
Diagnostic time (minutes)	23.5	25.5	0.60

## Data Availability

The original contributions presented in this study are included in the article. Further inquiries can be directed to the corresponding author(s).

## Abbreviations

CAD	coronary artery diseases
CABG	coronary artery bypass grafting
CTCA	computed tomography coronary angiography
ICA	invasive coronary disease
Stress ECG	stress electrocardiography
Stress ECHO	stress echocardiography
WMSI	wall motion score index
PCI	percutaneous coronary intervention
CTO	chronic total occlusion
NPV	negative predictive value
PPV	positive predictive value
MPS	myocardial perfusion scintigraphy
